# Fine‐scale habitat selection by sympatric Canada lynx and bobcat

**DOI:** 10.1002/ece3.6626

**Published:** 2020-08-17

**Authors:** Samantha J. Morin, Jeff Bowman, Robby R. Marrotte, Marie‐Josée Fortin

**Affiliations:** ^1^ Environmental & Life Sciences Graduate Program Trent University Peterborough ON Canada; ^2^ Ontario Ministry of Natural Resources & Forestry Wildlife Research & Monitoring Section Trent University Peterborough ON Canada; ^3^ Department of Ecology & Evolutionary Biology University of Toronto Toronto ON Canada

**Keywords:** bobcat, Canada lynx, habitat selection, *Lynx canadensis*, *Lynx rufus*, snow tracking

## Abstract

The Canada lynx (*Lynx canadensis*) and the bobcat *(Lynx rufus*) are closely related species with overlap at their range peripheries, but the factors that limit each species and the interactions between them are not well understood. Habitat selection is a hierarchical process, in which selection at higher orders (geographic range, home range) may constrain selection at lower orders (within the home range). Habitat selection at a very fine scale within the home range has been less studied for both lynx and bobcat compared to selection at broader spatiotemporal scales. To compare this fourth‐order habitat selection by the two species in an area of sympatry, we tracked lynx and bobcat during the winters of 2017 and 2018 on the north shore of Lake Huron, Ontario. We found that both lynx and bobcat selected shallower snow, higher snowshoe hare abundance, and higher amounts of coniferous forest at the fourth order. However, the two species were spatially segregated at the second order, and lynx were found in areas with deeper snow, more snowshoe hare, and more coniferous forest. Taken together, our findings demonstrate that the lynx and bobcat select different resources at the second order, assorting along an environmental gradient in the study area, and that competition is unlikely to be occurring between the two species at finer scales.

## Graphical abstract

We tracked Canada lynx and bobcats during the winters of 2017 and 2018 on the north shore of Lake Huron, Ontario, in order to compare their fourth‐order habitat selection. We found that both lynx and bobcats preferred more snowshoe hare and coniferous forest and avoided deeper snow at this scale, but were spatially segregated at the level of the second order. This indicates that despite similar limiting factors at the fourth order, limiting factors are likely different at broader scales and competition is likely not occurring at finer scales.

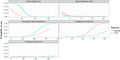

## 1 INTRODUCTION

Habitat selection is the behavioral process through which animals choose the resources that they use, and it is commonly measured as the use of a given resource relative to the availability of that resource in the surrounding environment (Manly, McDonald, Thomas, McDonald, & Erickson, 2002; Mayor, Schneider, Schaefer, & Mahoney, 2009). Habitat selection involves processes that are hierarchical, occurring at several orders or levels representing different spatiotemporal scales (Johnson, 1980): (a) the geographic range within the wider environment, (b) the home range within the geographic range, (c) habitat patches within the home range, and (d) fine‐scale selection within those patches. Habitat selection decisions made at larger spatial scales are usually made over longer periods of time (Bissonette, 1996; Holling, 1992; Wiens, 1989), and different resources may be selected at different scales (e.g., Anderson, 2005; Apps, McLellan, Kinley, & Flaa, 2001; Strickland, Villella, & Belant, 2016; VanderWerf, 1993). Processes within the home range (third‐ and fourth‐order selection) may be constrained by selection at the first and second orders (Rettie & Messier, 2000). Understanding how such constraints, or limiting factors, influence species distributions and habitat selection patterns has been promoted as a method for solving wildlife conservation problems (Morris, 2003) in a time when species worldwide are experiencing rapid anthropogenic change.

The Canada lynx (*Lynx canadensis*) is an iconic boreal specialist whose populations cycle with the snowshoe hare (*Lepus americanus*), its primary prey (O’Donoghue et al., 1998; Poole, 2003). The lynx has lost about 40% of its historical geographic range across North America (Laliberte & Ripple, 2004). In addition, there is evidence of a decline in both abundance and genetic diversity at the southern range edge (Bayne, Boutin, & Moses, 2008; Koen, Bowman, Lalor, & Wilson, 2014). There are several possible causes of the range contraction, including climate change (Koen, Bowman, Lalor, et al., 2014), a decline in snowshoe hare abundance (Murray, Steury, & Roth, 2008), and competition with other predators such as the coyote (*Canis latrans*) (Bunnell, Flinders, & Wolfe, 2006, Guillaumet, Bowman, Thornton, & Murray, 2015, but see Kolbe, Squires, Pletscher, & Ruggiero, 2007). Although classified as Least Concern by the IUCN (Vashon, 2016), the Canada lynx is listed as Threatened in the contiguous United States (USFWS, 2000) and as Endangered in Nova Scotia (Parker, 2001) and New Brunswick (New Brunswick Endangered Species Regulation, 2013). The lynx is adapted to traveling in snow with its large, furry feet (Buskirk, Ruggiero, Aubry, Pearson, & Squires, 2000) and selects areas with deep snow and snow cover at several spatial scales (Peers, Thornton, & Murray, 2013; Squires, Decesare, Kolbe, & Ruggiero, 2010).

A close relative of the Canada lynx, the bobcat (*Lynx rufus*), is more of a generalist whose geographic range spans from Mexico to southern Canada (Anderson & Lovallo, 2003). While the bobcat also commonly preys on lagomorphs, its alternative prey in northern regions include deer (*Odocoileus* spp.), squirrel, grouse, and porcupine (*Erethizon dorsatum*; e.g., Knick, Sweeney, Alldredge, & Brittell, 1984; Litvaitis, Clark, & Hunt, 1986; Matlack & Evans, 1992; McLean, McCay, & Lovallo, 2005). Over the past century, the range of the bobcat has been expanding northward into areas formerly and currently occupied by lynx (De Vos, 1964, Woolf & Hubert, 1998, Thompson, 2000, but see Gooliaff & Hodges, 2018a). Forest clearance for agriculture and urban development may be one reason for the expansion (Nowell & Jackson, 1996; Sunquist & Sunquist, 2002). The bobcat may be limited at its northern range edge by its high foot loading in deep snow; it has smaller paws than lynx and therefore lacks the lynx's ability to move and forage well in deep, soft snow (Buskirk et al., 2000; Parker, Maxwell, Morton, & Smith, 1983; Telfer & Kelsall, 1984). Indeed, it has been suggested that snow conditions historically limited bobcat invasion into areas occupied by lynx (Hoving, Joseph, & Krohn, 2003; Parker et al., 1983), and so a reduction in both the duration and depth of snow cover due to climate change may be contributing to the bobcat range expansion (Anderson & Lovallo, 2003; Peers et al., 2013). Climate change might therefore increase sympatry between lynx and bobcat, resulting in a higher frequency of encounters. For example, hybridization and backcrossing have been observed between the two species (Homyack et al., 2008; Koen, Bowman, Murray, & Wilson, 2014; Schwartz et al., 2004) and could potentially pose a threat to lynx in areas of increased sympatry (Koen, Bowman, Murray, et al., 2014).

Competitive interactions are another possible outcome of increased sympatry between lynx and bobcat. Interactions between the lynx and the bobcat are still not well understood, and competition may be contributing to the decline of the lynx at its southern range edge. The bobcat appears to be the more generalist of the two species as it has a larger niche breadth than the lynx (Peers, Thornton, & Murray, 2012). Parker et al. (1983) noted anecdotally that lynx densities declined on Cape Breton Island after colonization by bobcat. Peers et al. (2013) used bioclimatic distribution modeling to show niche displacement on a continental scale. Scully, Fisher, Miller, and Thornton (2018) compared occupancy and habitat associations of lynx, bobcat, and cougar in northern Washington, and found a decrease in the use of camera sites by lynx in northern Washington when bobcats were present. However, it remains unclear whether lynx are directly displaced due to competition with bobcats, or bobcats simply occupy vacated areas after lynx have retreated due to other factors.

Habitat selection by the Canada lynx and the bobcat has not been directly tested and compared in an area where they co‐occur. However, the single‐species occupancy models from Scully et al. (2018) showed that the use by lynx was associated with both abiotic and hare covariates, while the use by bobcat was only associated with abiotic factors. They also found that spatial overlap of lynx and bobcats was greater in snow‐off versus snow‐on seasons, potentially indicating avoidance of deep snow by bobcat. Studies of selection by lynx at a landscape level and within home ranges generally have found a preference for younger coniferous forests (e.g., Fuller, Harrison, & Vashon, 2007; Hoving, Harrison, Krohn, Jakubas, & McCollough, 2004; Mowat & Slough, 2003), likely because regenerating and immature forest stands are denser and have branches closer to the ground, therefore providing better snowshoe hare habitat. Bobcats prefer a variety of different land cover types, often several within the same study area, including coniferous forest, deciduous forest, wetland, and grassland (e.g., Kamler & Gipson, 2000; Reed et al., 2017; Tucker, Clark, & Gosselink, 2008), although resources that have been preferred in some studies have been avoided in others and vice versa. Habitat selection at a very fine scale within the home range (fourth‐order selection), which represents foraging and other daily movements, has been less studied for both species compared to selection at broader spatiotemporal scales (but see Anderson, 1990; Organ et al., 2008; Squires, Decesare, Kolbe, & Ruggiero, 2008; Squires et al., 2010).

Here, we directly compare fourth‐order habitat selection by the Canada lynx and the bobcat in an area of sympatry. We aimed to determine the resources that are important to each species when measured at a fine spatial scale. This is the first comparison of fine‐scale resource selection by the two species at the confluence of their ranges. We hypothesized that the lynx is a boreal forest and snowshoe hare specialist and the bobcat is a generalist limited by snow conditions. We predicted that the lynx would prefer higher densities of snowshoe hare and forest stands with higher amounts of coniferous forest and immature forest, while the bobcat would show preference for alternative prey species in addition to snowshoe hare, as well as avoidance of deep and soft snow.

## 2 METHODS

### 2.1 Study area

**Figure 1:**
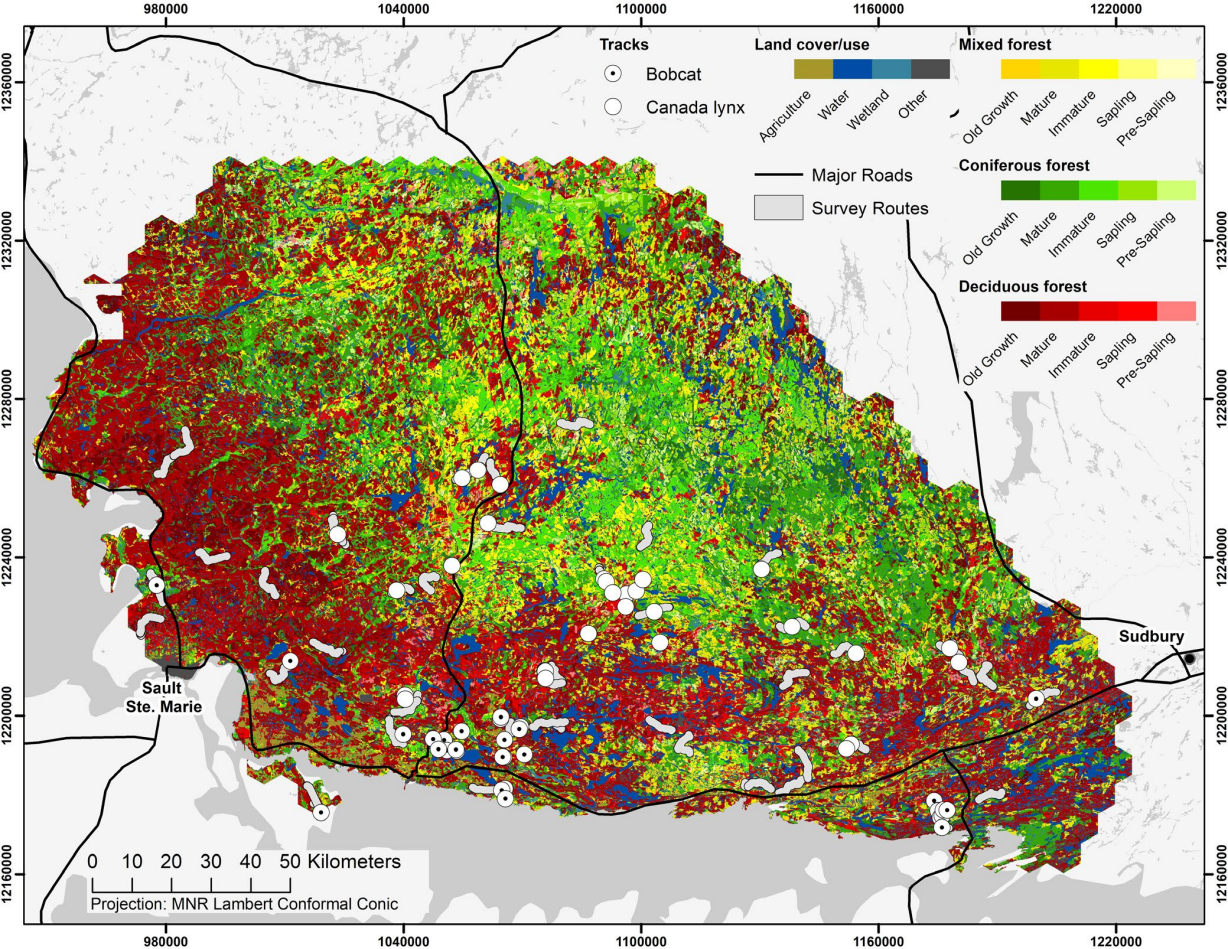
Locations of snow‐tracked Canada lynx (*Lynx canadensis*) and bobcat (*Lynx rufus*), January–March 2017 and 2018, on the north shore of Lake Huron, Ontario. Empty circles represent locations where lynx were tracked, and dotted circles represent locations where bobcats were tracked. Gray lines represent snowmobile routes used to survey for tracks, 1–3 times per winter. Black lines represent major roads. Colors in the landscape represent different forest types, as well as other land cover and land use types

Fur harvest records demonstrate that there is an area of Canada lynx and bobcat range overlap on the north shore of Lake Huron in Ontario, Canada. Ontario Ministry of Natural Resources and Forestry (OMNRF) records show that between 2000 and 2009, almost all bobcats harvested in Ontario were trapped between Sudbury and Sault Ste. Marie. Our study area (Figure 1), covering approximately 33,000 km^2^, was based on the 75% minimum convex polygon of these bobcat trapping records (buffered by 35 km to accommodate nearby traplines or townships). The land cover was 37.6% deciduous forest, 29.3% coniferous forest, 14.3% mixed forest, 9.5% water bodies, 5.1 wetlands, and 1.1% agriculture, with the remaining 3.0% including mines, outcrops, and urban areas. The region encompassed a transition between the Great Lakes‐St. Lawrence forest of central Ontario, and the boreal forest of northern Ontario. The Great Lakes‐St. Lawrence forest is dominated by deciduous trees but also includes some coniferous species. The boreal forest is characterized by predominantly coniferous species with mixed‐wood stands. Overall, the landscape had been influenced by forest fires, past and current logging and forest management, hydroelectric development, and agricultural activities to some extent.

### 2.2 Snow tracking

We evaluated fine‐scale habitat selection by Canada lynx and bobcat in winter by comparing track segments of each species to available segments in the immediate vicinity. We therefore conducted snow tracking of Canada lynx and bobcat throughout the study area from January to March 2017 and 2018. We used snowmobile survey routes to search systematically for tracks, which we then followed in order to measure snow, prey, and forest characteristics. In addition to these systematic surveys, we also sampled tracks observed opportunistically as we traveled through the study area. We used a paired design of sampling tracks and available habitat adjacent to tracks (see below), so inclusion of opportunistic samples did not bias our fine‐scale comparison of used and available habitat. The effort for opportunistic tracking was evenly distributed throughout the study area, as we consistently scanned for tracks while traveling.

To determine where we would survey, we followed rules established by a contemporaneous collaborative study occurring in the same area (Marrotte, Bowman, & Morin, 2020). The study area was divided into hexagonal sampling units of 64 km^2^. We based this size on previous lynx snow tracking studies that have used sampling units of 8 × 8 km or 64 km^2^ (e.g., Squires, Olson, Turner, DeCesare, & Kolbe, 2012). The hexagons to be surveyed were selected using a clustering algorithm based on their associated land cover and land use composition. Forest Resource Inventory (FRI; OMNRF, unpublished data) maps were used to categorize each forest stand by forest type and by seral stage. Additional areas that were not forest stands were classified as agriculture, water, wetland, and other (all other land cover types). To choose which hexagons would be surveyed, affinity propagation (Frey & Dueck, 2007) was used to extract the proportion of each class within each hexagon and then cluster the hexagons into groups characterized by different compositions of land cover and land use. For each of the groups, the exemplar (most representative hexagon of the group) was surveyed as well as hexagons crossed to reach the exemplar. See Marrotte et al. (2020) for more details about site selection.

Survey routes varied in length from 7 to 10 km. Squires et al. (2012) found that after 7 km of searching for lynx tracks, the probability of detection reached an asymptote. We surveyed each route at least once and up to three times each winter. We waited 24–48 hr after fresh snowfall before surveying, in order to allow for track accumulation (Maletzke, Koehler, Wielgus, Aubry, & Evans, 2008; Potvin, Bertrand, & Ferron, 2005). Additionally, we only proceeded with tracking when snow conditions were good (minimal wind drift, debris, ice, or melting), allowing for track dimensions and species‐specific characteristics to clearly be identified. The tracks of these two species are distinct; lynx tracks are larger than bobcat tracks and show more fur. Bobcats have hairless footpads, whereas lynx do not (Elbroch, 2003).

We omitted the first 50 m of a discovered track to avoid taking misleading measurements at the road or trail edge where snow may have been piled by passing vehicles, and to reduce the systemic bias introduced by always searching for tracks on trails and roads. Starting at the 50‐m mark, we retraced the animal's path for 500 m while continuously recording the track on a GPS unit.

We preferred back‐tracking to avoid following the animal and influencing behavior; however, forward‐tracking was sometimes required instead due to poor snow conditions, impassable terrain, private property to which we had not been granted access, or lost tracks. In cases when back‐tracking could not be continued for such reasons, but 500 m had not yet been completed, we returned to the starting point and added the remaining distance by forward‐tracking. When the tracks belonged to a family unit (i.e., a female with one or more kittens), we followed the female's tracks if hers split from those of her offspring.

We took snow depth and hardness measurements at the beginning of the 500 m and every 100 m thereafter, for a total of six sets of snow measurements per path. Measurements were taken immediately beside the track closest to the 100‐m mark. We measured snow depth from the top of the crust to the ground surface, using a metal metre stick and digging to ground level if necessary. We measured snow hardness as the depth in centimeters penetrated by a 150‐g plastic ball, 5 cm in diameter, when dropped from 1 m above the snow surface. A larger magnitude of penetration therefore indicates softer snow. We counted all other individual animals that intersected the lynx or bobcat path (identifying the species where possible). White‐tailed deer or hare trails showing frequent use, where it was impossible to distinguish individual tracks, were arbitrarily counted as five tracks. An arbitrary value is typical for this sort of problem. For example, Thompson, Davidson, O'Donnell, and Brazeau (1989) used an arbitrary value of 4 tracks for hare trails.

After following the animal's “used” path for 500 m, we immediately took measurements of a corresponding “unused” path in a similar way. We moved 100 m away from the end of the used path randomly either to the left or to the right as decided with a coin toss, and used the GPS unit to walk 500 m parallel to the used path—creating a path with the same profile as the used path but 100 m away. This scale was in part based on previous literature, although there are few studies of fourth‐order selection by either species. 100 m was one of three scales used by Squires et al. (2008) to analyze fourth‐order lynx den site selection, and it was also the maximum distance set by Anderson (1990) between used and random bobcat daytime resting sites. We took another six sets of snow measurements (measuring immediately beside the footprint closest to each 100‐m mark) and counted all other animals’ tracks that we crossed. If we encountered further lynx or bobcat tracks that could have been made by the same individual followed for the used path, we moved another 100 m away and tried again to create a path that had not been used. Similar to the used path, if for any reason we could not continue, we went back to the starting point and added the remaining distance in the other direction. Measurements of each unused path were usually completed within an hour of beginning the corresponding used path.

### 2.3 Statistical analysis

Because we did not know the scale at which the lynx and bobcats were perceiving their environment, we created a multiple ring buffer around each path in ArcMap 10.6 (ESRI, 2017), with buffer sizes of 10, 20, 30, 40, and 50 m. We set a 10 m minimum because it reflected the general error of Garmin GPS location accuracy, while 50 m was the maximum because a buffer any larger would overlap with buffers for the other path in the pair (as used and unused paths were 100 m apart). Using the FRI polygons, for each buffer size we extracted the proportion of each path made up of forest (versus nonforest land cover types), the percentages of coniferous forest in the forest stands covered by each path, and the percentages of immature forest in the forest stands covered by each path. We defined “immature forest” as all nonmature seral stages as classified by the FRI: presapling, sapling, and immature.

To uncover patterns of habitat selection for each species, we performed model selection based on the snow conditions and major prey species data collected in the field and the forest attributes. We first performed conditional logistic regressions using the "clogit" function in the *survival* package (Therneau, 2015) in R (version 3.5.1, R Core Team, 2018) for the full model at each scale, with the used (1) or unused (0) responses as paired comparisons. Our models assumed that the pairs of used and unused paths were independent. The following predictors were included in the full models: snow depth (cm) and snow hardness (cm) averaged from the six measurements; the relative abundances of snowshoe hare (tracks/km), deer (tracks/km), squirrel (tracks/km), and grouse (tracks/km); and the landcover composition based on coniferous forest (%) and immature forest (%). We compared the AICc of the full models for each buffer size to determine which scale had the best fit. We compared the mean of each variable between lynx and bobcat for both used and unused paths, using the coniferous forest and immature forest amounts at the scale with the best fit. We also created a correlation matrix for the Pearson correlations between the amounts of coniferous forest at the different buffer scales, and a matrix for the correlations between the amounts of immature forest at each scale.

Before creating the resource selection models for the fourth order, we compared the mean of each variable between lynx and bobcat paths, both used and unused, using the forest attributes from the buffer scale with the best fit. We did this without considering the paired nature of the paths, to examine general, broader‐scale differences between habitat conditions associated with each species.

We then proceeded to model habitat selection at the fourth order. For the buffer scale with the best fit for the full model, we checked the correlations between each predictor variable, as we suspected that snowshoe hare abundance may have depended on some of the other environmental conditions. We also tested for an effect of the sampling year before proceeding by running both a univariate logistic regression with year (2017 or 2018) as the predictor, and by running a full model with all variables plus year. We then used AICc to select the best model out of five candidate models with different combinations of the categories of habitat predictors: (a) the full model of snow (depth and hardness), hare (snowshoe hare), alternative prey (deer, squirrel, and grouse), and forest (coniferous and immature); (b) hare and forest; (c) snow, hare, and forest; (d) hare, alternative prey, and forest; and (5) snow, hare, and alternative prey.

Given the boreal specialist nature of the Canada lynx, we predicted that lynx paths would be positively associated with hare activity and boreal forest stand types. In contrast, given the generalist nature of the bobcat, we predicted that bobcats would be associated with more heterogeneous land cover and prey.

After determining the top models for lynx and for bobcat, we then used those models to predict the probability of use by each species over the range of each resource variable. When doing this for each variable, we set the other covariates to their mean values.

## 3 RESULTS

We obtained 30 pairs of used and unused paths for both Canada lynx and bobcat (Figure 1). Lynx and bobcat tracks were never encountered on the same survey route. In general, bobcats were encountered and tracked closer to the shore of Lake Huron and toward the western side of the study area, whereas lynx were farther north (Figure 1). Lynx tracks represented 21 different hexagons with 1–3 tracks per hexagon, and bobcat tracks represented 14 different hexagons with 1–6 tracks per hexagon.

When looking at the paths in general, without considering their paired nature, we found tracks of snowshoe hare and squirrel (red, *Tamiasciurus hudsonicus*; or northern flying, *Glaucomys sabrinus*) much more frequently on Canada lynx paths (both used and unused) than any other species (Table 1). For bobcat paths, white‐tailed deer tracks were also found most frequently along with snowshoe hare and squirrel. Species that intersected one or more bobcat paths but no lynx paths were porcupine, raccoon (*Procyon lotor*), and wild turkey (*Meleagris gallopavo*). Overall, 10% of deer tracks and 12% of hare tracks came from the “well‐used trail = 5 tracks” rule for lynx paths, while 9% of deer tracks and 15% of hare tracks came from this rule for bobcat paths.

**Table 1:** Mean (±SE) number of other species tracks per kilometer found on used and unused paths for Canada lynx (*Lynx canadensis*) and bobcat (*Lynx rufus*) on the north shore of Lake Huron, Ontario

	Canada lynx		Bobcat	
	Used	Unused	Used	Unused
Snowshoe hare	39.07 ± 5.23	25.04 ± 3.46	20.02 ± 4.07	20.14 ± 4.11
Squirrel	10.12 ± 1.98	9.00 ± 1.61	7.96 ± 2.04	7.38 ± 1.89
Deer	0.19 ± 0.14	0	10.19 ± 2.71	9.64 ± 2.46
Grouse	0.71 ± 0.26	0.51 ± 0.17	0.50 ± 0.23	0.94 ± 0.42
Moose	0.26 ± 0.19	0.07 ± 0.07	1.08 ± 0.98	1.04 ± 0.55
Wolf or coyote	0.57 ± 0.26	0.40 ± 0.17	1.81 ± 0.66	1.71 ± 0.47
Red fox	0.15 ± 0.11	0.13 ± 0.13	0.39 ± 0.19	0.22 ± 0.13
Weasel	0.58 ± 0.24	0.37 ± 0.22	0.16 ± 0.13	0.64 ± 0.33
Fisher	0.17 ± 0.09	0.53 ± 0.24	0.29 ± 0.15	0.41 ± 0.23
American marten	0	0.12 ± 0.12	0	0.06 ± 0.06
River otter	0	0.87 ± 0.52	0.07 ± 0.07	0.19 ± 0.14
Mouse	0.54 ± 0.35	0.38 ± 0.27	0.93 ± 0.48	1.15 ± 0.86
Vole or shrew	0.05 ± 0.05	0.40 ± 0.20	0.57 ± 0.38	1.06 ± 0.60
Porcupine	0	0	0.40 ± 0.28	0
Raccoon	0	0	0	0.04 ± 0.04
Wild turkey	0	0	0	0.49 ± 0.49

Note

For both Canada lynx and bobcat used and unused paths, *n* = 30.

**Figure 2:**
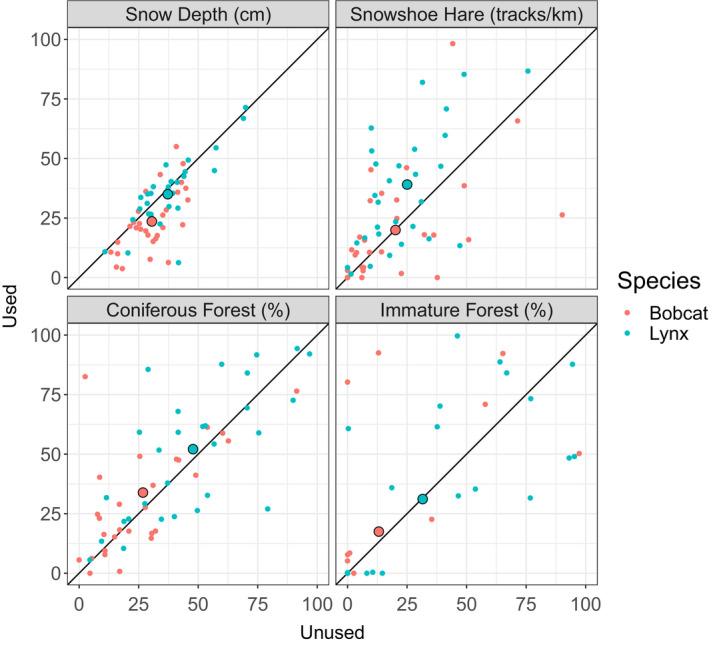
Snow depth (cm), snowshoe hare (tracks/km), coniferous forest (%), and immature forest (%) on unused versus used Canada lynx (*Lynx canadensis*) and bobcat (*Lynx rufus*) paths on the north shore of Lake Huron, Ontario. Blue dots represent Canada lynx, and red dots represent bobcat. The solid line represents a 1:1 relationship. The larger points represent the mean for each species

In general, Canada lynx paths had deeper snow (*t* = 3.86, *p* < .001; Table 2, Figure 2), softer snow (*t* = 2.43, *p* < .05; Table 2), more snowshoe hare tracks (*t* = 2.78, *p* < .001; Table 2, Figure 2), higher percentages of coniferous forest (*t* = 4.15, *p* < .001; Table 2, Figure 2), and higher percentages of immature forest (*t* = 2.60, *p* < .05; Table 2, Figure 2) than bobcat paths. Bobcat paths had more deer tracks (*t* = 5.41, *p* < .001), whereas the numbers of squirrel and grouse tracks were not different between the two species (Table 2).

**Table 2:** Mean (±SE) snow depth, snow hardness, number of snowshoe hare tracks, number of other prey species tracks (deer, squirrel, and grouse), amount of coniferous forest, and amount of immature forest for used and unused Canada lynx *(Lynx canadensis*) and bobcat (*Lynx rufus*) paths on the north shore of Lake Huron, Ontario

	Canada lynx		Bobcat	
	Used	Unused	Used	Unused
Snow depth (cm)	40.0 ± 2.66	37.2 ± 2.40	23.6 ± 2.36	30.5 ± 1.75
Snow hardness (cm)	10.6 ± 0.94	11.1 ± 0.95	7.9 ± 0.89	9.2 ± 1.04
Snowshoe hare (tracks/km)	39.1 ± 5.23	25.0 ± 3.46	20.0 ± 4.07	20.1 ± 4.11
Deer (tracks/km)	0.19 ± 0.15	0	10.2 ± 2.71	9.65 ± 2.46
Squirrel (tracks/km)	10.1 ± 1.98	9.00 ± 1.61	7.96 ± 2.04	7.38 ± 1.89
Grouse (tracks/km)	0.71 ± 0.26	0.51 ± 0.17	0.50 ± 0.23	0.94 ± 0.42
Coniferous forest (%)	52.1 ± 5.27	47.8 ± 4.70	33.9 ± 4.78	26.8 ± 4.02
Immature forest (%)	31.1 ± 6.45	31.6 ± 6.68	17.5 ± 6.02	13.1 ± 5.58

Note

Snow hardness was measured as the depth penetrated below the surface of the snow, and therefore, a larger snow hardness value indicates softer snow. The amounts of coniferous forest and immature forest were calculated with a 20‐m buffer around the paths. For both Canada lynx and bobcat used and unused paths, *n* = 30.

The full models of habitat selection using a 20‐m buffer had the best fit for both Canada lynx and bobcat, as evidenced by the lowest AICc among the five models tested for each species (Table S1). The amounts of coniferous forest were highly correlated between the five buffer scales for both lynx and bobcat (*r* ≥ 0.946; Table S2) as were the amounts of immature forest (*r* ≥ 0.952; Table S3). At the 20‐m buffer size, none of the variables were highly correlated with any other variables, with │*r*│ ≤ 0.379 for lynx and │*r*│ ≤ 0.459 for bobcat (Table S4). We did not find an effect of year either as the sole predictor or when included with all other variables. Of the five candidate models of habitat selection that were then tested at the 20‐m buffer scale, the best model for Canada lynx and also for bobcat was model 3, which included snow depth, snow hardness, snowshoe hare, coniferous forest, and immature forest as predictors. For both species, model 3 had the lowest AICc values, highest Akaike weights (0.539 for lynx and 0.969 for bobcat), and the highest pseudo *r* ^2^ among models with the same number of predictors or fewer (Table 3). These models performed better than the null models, as the reductions in deviance from null to residual (20.61 for lynx, 22.74 for bobcat) were much greater than the number of parameters (6) in the top models.

**Table 3:** Model selection results from conditional logistic regression of the effects of snow conditions, prey species, and forest attributes on the likelihood of path use by Canada lynx (*Lynx canadensis*) and bobcat (*Lynx rufus*)

Species	Predictors	*K*	AICc	ΔAICc	AICc weight	*R* ^2^
Lynx	Snow + Hare + Forest	6	32.09	0	0.539	0.496
	Snow + Hare + Alternative Prey	7	34.43	2.34	0.167	0.499
	Hare + Forest	4	34.91	2.82	0.132	0.315
	Snow + Hare + Alternative Prey + Forest	9	35.07	2.98	0.122	0.609
	Hare + Alternative Prey + Forest	7	37.25	5.16	0.041	0.431
Bobcat	Snow + Hare + Forest	6	29.96	0	0.969	0.566
	Snow + Hare + Alternative prey + Forest	9	39.86	9.90	0.025	0.577
	Snow + Hare + Alternative prey	7	41.26	11.30	0.005	0.384
	Hare + Forest	4	43.14	13.18	0.001	0.099
	Hare + Alternative prey + Forest	7	49.16	19.20	0.000	0.166

Note

*K* is the number of model parameters. AICc is the Akaike information criterion corrected for small sample size, ΔAICc is the difference in AICc between each model and the top model, AICc weight indicates the likelihood of the model being the best model given the overall model set, and *R* ^2^ is McFadden's pseudo *R* ^2^. Snow includes snow depth and snow hardness; hare is snowshoe hare; alternative prey includes deer, squirrel, and grouse; and forest includes coniferous forest and immature forest.

Based on the lynx best model for the 20‐m scale, lynx selected for lower snow depth, higher densities of snowshoe hare, and higher amounts of coniferous forest (Table 4). Based on the bobcat best model, bobcats showed the same selection as lynx, but also avoided softer snow. For snow depth, hare, and coniferous forest, the magnitude of the coefficient estimates did not differ between lynx and bobcat when standard errors were compared (Table 4).

**Table 4:** Parameter estimates (±SE) from the best models of Canada lynx (*Lynx canadensis*) and bobcat *(Lynx rufus*) habitat selection on the north shore of Lake Huron, Ontario

Species	Snow depth (cm)	Snow hardness (cm)	Snowshoe hare (tracks/km)	Coniferous forest (%)	Immature forest (%)
Canada lynx	−0.260 ± 0.138	−0.041 ± 0.374	0.099 ± 0.038	6.046 ± 3.442	−0.045 ± 3.436
Bobcat	−0.219 ± 0.105	−0.505 ± 0.392	0.065 ± 0.033	12.597 ± 8.106	2.403 ± 4.923

Note

Snow hardness was measured as the depth penetrated below the surface of the snow, and therefore, a larger snow hardness value indicates softer snow. Parameter estimates represent the influence on the log‐odds of path use for a one‐unit increase in that variable.

**Figure 3:**
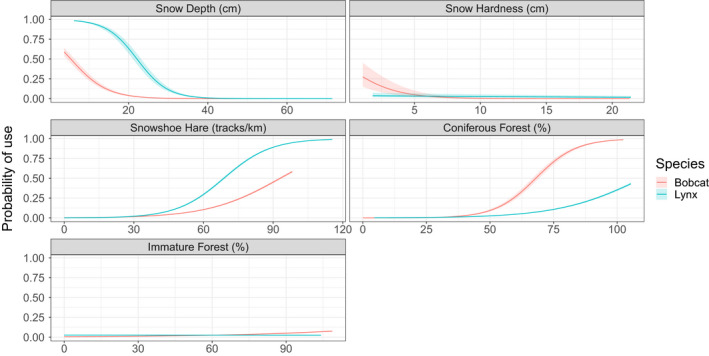
Predicted probability of use by Canada lynx and bobcat for snow depth, snow hardness, snowshoe hare, coniferous forest, and immature forest. Probabilities were predicted from conditional logistic regressions of used and unused paths. Red represents bobcat, and blue represents Canada lynx. Shaded areas represent the 95% confidence interval

The predicted probability of use increased with increasing snowshoe hare and coniferous forest, and decreased with increasing snow depth, for both lynx and bobcat (Figure 3). Bobcat use also decreased with softer snow. Overall probability of use was lower for bobcat than for lynx over the range of snow depths used to predict, reaching a probability of zero at about 20 cm versus 40 cm for lynx. The probability of use increased at a faster rate for lynx than for bobcat with increasing snowshoe hare after approximately 30 tracks/km. Conversely, bobcat probability of use of coniferous forest increased at a faster rate than for lynx after approximately 35%.

## 4 DISCUSSION

### 4.1 Second‐order selection

The spatial segregation and habitat differences we observed between lynx and bobcat in our study area suggests that there is little, if any, overlap between the two species at the second order, at least during the winter season. These kinds of differences were also found by Marrotte et al. (2020) in our study area. Lynx generally occurred in areas with deeper snow as well as more hare, coniferous forest, and immature forest. Bobcat used and unused paths had more tracks of species associated with typically more southern, mixed‐wood or open habitats (e.g., white‐tailed deer, raccoon, wild turkey), although they also had many more moose and canids. It appears that the lynx and bobcat likely select different resources at the second order within our study area, and it is important to note that these preferences may affect the finer‐scale selection discussed in the following section. As land use in the matrix of a landscape can influence the use of habitat patches (e.g., Da Silva, Ribeiro, Hasui, da Costa, & da Cunha, 2015; Lesmerises, Ouellet, Dussault, & St‐Laurent, 2013; Rodewald, 2003), lynx may be less likely to use suitable habitat patches at the second order due to human influence on the landscape in the southern part of the study area.

Combined with the distribution of bobcats mainly along the shore in the south and lynx toward the interior, our results suggest that lynx and bobcats are assorting along a north–south gradient in our study area similar to that of several mammal species found by Bowman, Ray, Magoun, Johnson, and Dawson (2010) in northwestern Ontario. In another area of broad distribution overlap between lynx and bobcat in southern British Columbia, Gooliaff, Weir, and Hodges (2018b) found that the two species were generally segregated at a finer scale based on elevation, with lynx at higher elevations and bobcat at lower elevations. The occupancy models of Scully et al. (2018) showing decreased lynx–bobcat overlap in snow‐on seasons also support the importance of snow as a limiting factor for bobcats, and the overall role of bioclimatic conditions in limiting lynx–bobcat interactions. Along with our results, these studies suggest that home range allopatry may occur throughout lynx–bobcat range overlap across North America.

### 4.2 Fourth‐order selection

Our analysis of used and unused Canada lynx and bobcat paths produced mixed support for our predictions of fine‐scale habitat selection by the two species. Based on previous descriptions of the lynx and the bobcat as a specialist and a generalist, respectively, we expected to see some overlap in selected resources, but also some differences. However, we found that the two species select the same set of resources when measured at the fourth order as we have defined it here (i.e., 100‐m scale). Both avoided deeper snow and preferred higher densities of snowshoe hare and greater amounts of coniferous forest.

The avoidance of deeper snow by lynx was somewhat surprising. It is possible that lynx show indifference or preference for snow depth at broader scales, but prefer to travel in patches of relatively shallower snow during their daily movements. This would make sense given that lynx have been shown to travel on trails and roads (Moen, Terwilliger, Dohmen, & Catton, 2010; Mowat & Slough, 2003). Also, the overall greater probability of use of snow depth by lynx compared to bobcat is consistent with the idea that lynx are better adapted to deeper snow. In general, there is likely a lower threshold of snow depth avoidance for bobcat than for lynx across several orders of selection, given their higher foot loading (Buskirk et al., 2000; Parker et al., 1983; Telfer & Kelsall, 1984).

The lack of selection by lynx for immature forest was also unexpected at the fourth order of selection. Because our method of snow tracking and subsequent analyses did not attempt to differentiate between different kinds of use, such as foraging or territory marking, it could be that lynx select different seral stages for different activities or at different times of day. A congeneric species, the Eurasian lynx (*Lynx lynx*), has been shown to select dense cover during the day to avoid human activity, and open habitat at night in order to hunt its ungulate prey (Filla et al., 2017). For foraging specifically, snowshoe hare may be more abundant in younger coniferous forest stands, but visibility and access by lynx may be greater in more mature stands (Fuller et al., 2007; Ivan & Shenk, 2016). Some studies have found that lynx prefer mature forest in addition to immature forest, depending on sex and season (e.g., Holbrook, Squires, Olson, DeCesare, & Lawrence, 2017; Squires et al., 2010; Vashon et al., 2008).

In our analysis, snowshoe hare was not highly correlated with coniferous forest or immature forest, indicating that there could be additional factors influencing habitat selection by hare and therefore also by Canada lynx. Snowshoe hares have been shown to use edge habitat (e.g., Gigliotti, Jones, Lovallo, & Diefenbach, 2017; Mowat & Slough, 2003; Pietz & Tester, 1983), which in our study could have translated to fewer hare tracks inside forest stands. While we could have expected that paths with higher snowshoe hare abundance were more likely to be used by lynx than by bobcat after a certain threshold, we did not expect that paths with higher amounts of coniferous forest would have a higher predicted probability of use by bobcat than by lynx. This is surprising, but it could be partially explained by the importance of snowshoe hare to the lynx and a lack of correlation between hare and coniferous forest.

We would like to reiterate that in the hierarchical framework of habitat selection, more important limiting factors are selected for at higher orders (Rettie & Messier, 2000), so it is possible that selection for immature forest by lynx at the second or third order has resulted in sufficient abundance of young forest stands that further selection is not necessary at the fourth order. Similarly, it could be that selection for factors other than coniferous forest was more important for bobcat at the second and third orders, leading to a preference for coniferous forest at the fourth order that the lynx has already met at higher orders. This concept of trade‐offs between resources, as well as thresholds for preference or avoidance of habitat, refers to functional responses in habitat selection. Functional responses, that is, selection varying with the availability of a given resource (Mysterud & Ims, 1998), could help explain the unexpected results for the variables described above.

We had expected to find a preference by bobcat for the three alternative prey species in addition to a preference for snowshoe hare. That we did not find this could mean that the bobcat has become more specialized on snowshoe hare at its northern range edge, while only killing deer, squirrel, and grouse opportunistically. If hare are locally more abundant than other prey, this could reflect a high degree of plasticity in bobcat behavior as a generalist predator (Tuomainen & Candolin, 2011). It is quite possible that the bobcat is a “facultative generalist” (Shipley, Forbey, & Moore, 2009), having a broad fundamental niche but displaying a narrow realized niche in certain populations. Another possibility is that the bobcat has not specialized on hare, but is preying on hare in addition to species that we did not consider as potential major diet components based on the literature for bobcats in eastern North America, or that we did not include in the analysis because of low occurrence on paths. The one bobcat kill site we observed was a porcupine, but we found porcupine tracks very rarely on bobcat paths. Newbury and Hodges (2018) found that in Montana at the northern edge of the bobcat range, bobcats consumed mainly red squirrels and rodents, while hare, deer, and grouse comprised only a small part of the dietary biomass. However, their analysis of bobcat diet studies conducted in northern populations across North America showed that bobcats in eastern populations consumed more lagomorphs and deer than western populations. More research is needed to investigate bobcat prey selection in areas of overlap with lynx.

### 4.3 Limitations and scope

There are several caveats to our analysis. First, the results may have been affected by the way in which paths were sampled, while we strove for an unbiased sampling design by searching for tracks in sampling units representing the full range of habitat conditions available in the study area, accessibility, terrain, and human activities affected where we could actually complete snow tracking. We were unable to consider the effect of individual selection, due to our visual method of track identification. Including DNA analysis of hair and scat (McKelvey et al., 2006) or eDNA analysis from the tracks themselves (Franklin et al., 2019) could have allowed us to identify individuals. Determining the average home range size in our study area through telemetry could have provided a rough estimate of the maximum number of individuals considered in this study, but the cost and effort required to collar enough of each species would have been logistically challenging.

It is difficult to say why the lynx would avoid deep snow but be apparently indifferent to snow hardness, with virtually no change in the predicted probability of the use over a range of snow hardness values. It could be that snow hardness is not as meaningful at this scale. Due to wind, terrain, and overhead cover, snow depth can vary significantly over relatively small distances as we observed during this study, but since hardness is linearly correlated with air temperature (Tusima, 1975), it may be less likely to vary over 100 m. Also, our observations may not have represented the same conditions as when the tracks were made. It is difficult to evaluate snow hardness as a useful indicator since we did not compare the actual sinking depth of the lynx and bobcat. Sinking depth has been shown to be an important limiting factor for mammals (e.g., Halpin & Bissonnette, 1988; Murray & Boutin, 1991; Ossi, Gaillard, Hebblewhite, & Cagnacci, 2015; Parker, Robbins, & Hanley, 1984).

We considered prey species in our analyses but did not consider other carnivores, such as coyotes and mustelids, which were common in both lynx and bobcat areas. The presence of these species may have influenced lynx and bobcat habitat selection. Evidence for competition between coyotes and bobcats is mixed (e.g., Fedriani, Fuller, Sauvajot, & York, 2000; Litvaitis & Harrison, 1989; Neale & Sacks, 2001; Thornton, Sunquist, & Main, 2004), and although limited in deep snow, coyotes may be able to access Canada lynx habitat through the use of snowmobile trails (Bunnell et al., 2006, but see Kolbe et al., 2007). While smaller weasels are likely not in competition with lynx and bobcats for their larger prey items, the snowshoe hare is an important prey species for the fisher (*Pekania pennanti*; Arthur, Krohn, & Gilbert, 1989; Bowman, Donovan, & Rosatte, 2006; Raine, 1987), and a mesopredator release has been documented for fishers in areas where their range has expanded (LaPoint, Belant, & Kays, 2015).

While we found that a 20‐m path buffer provided the best model fit, it is unlikely that lynx and bobcats perceive and respond to their environment at a constant scale while moving. Researchers have historically considered that an animal's window of detection of the landscape (its perceptual range, Lima & Zollner, 1996) is static, but this assumption has ignored the context dependence of environmental factors that can modify this range (Olden, Schooley, Monroe, & Poff, 2004). In some cases, the plasticity of perceptual range is substantial (e.g., Schooley & Wiens, 2003). In our study, however, coniferous forest measures were highly correlated across all scales, as were immature forest measures, implying that model results would likely not differ substantially had a different buffer size been used for both variables, or had different buffer scales for each variable been combined.

It is also important to note that these results give limited insight into the process of resource competition between the two species. With respect to the observed second‐order segregation, it could be that we are seeing the result of niche displacement having already occurred at the level of the home range (the “ghost of competition past” Connell, 1980), although this effect would be more likely if there was a longer term equilibrium between the two species. The alternative is that different habitat selection at the home range level is mediating coexistence and broadscale sympatry. Yet, because habitat selection is generally viewed as a hierarchical process (Johnson, 1980), it is unlikely that competition has occurred at a fine scale between the lynx and the bobcat despite the similar fourth‐order habitat selection. Constraints may occur at a higher order, reinforcing allopatry.

### 4.4 Future research directions

Since we did not compare fourth‐order selection for behaviors associated with den sites, kill sites, or resting areas, we suggest that additional studies should be conducted at this level, as well as at higher levels, to complete the picture of habitat selection by the two species specifically in areas of range overlap. Multiscale studies provide better characterization of habitat use patterns than studies conducted at single scales (Poizat & Pont, 1996), and selection cannot always be reliably extrapolated across scales (Schneider, 1994; Wiens, 1989). To elucidate competitive interactions, selection by the two species should be compared for environmentally similar areas of allopatry and sympatry.

Telemetry or GPS studies would provide more definitive answers about home range size and overlap, and would allow for analysis of the effects of sex, age, and individuality on selection. These results represent winter habitat selection, and it is possible that different resources are selected in other seasons as shown by studies such as Chamberlain, Leopold, and Conner (2003) and Squires et al. (2010), or that the results we observed are remnants of decisions or constraints imposed by other seasons or even previous years. Studies of multilevel habitat selection by both species using GPS collars could investigate the seasonality of selection in sympatry. We recommend further assessing interactions between these two species, other carnivores, and snowshoe hare. Additionally, future research should further investigate snow conditions, as snow has previously been neglected in most lynx and bobcat habitat selection studies and appears, from our findings, to be important in their resource selection.

## 5 CONCLUSION

This was the first study comparing fine‐scale habitat selection by the Canada lynx and the bobcat in an area of range overlap, and our results indicate that they show similar habitat selection at the fourth order. Understanding the resources that are preferred and avoided by lynx and bobcat at all scales is important for predicting future range limit changes for both species, providing context for hybridization and other biotic interactions, and planning future conservation efforts for the lynx.

## CONFLICT OF INTEREST

The authors declare that there are no conflicts of interest.

## AUTHOR CONTRIBUTION

**Samantha J Morin:** Conceptualization (equal); Data curation (lead); Formal analysis (lead); Funding acquisition (equal); Writing‐original draft (lead); Writing‐review & editing (lead). **Jeff Bowman:** Conceptualization (equal); Funding acquisition (equal); Project administration (lead); Supervision (lead); Writing‐original draft (supporting); Writing‐review & editing (supporting). **Robby Marrotte:** Conceptualization (equal); Data curation (supporting); Formal analysis (supporting); Funding acquisition (equal); Writing‐original draft (supporting); Writing‐review & editing (supporting). **Marie‐Josée Fortin:** Conceptualization (equal); Funding acquisition (equal); Supervision (supporting); Writing‐original draft (supporting); Writing‐review & editing (supporting).

### OPEN RESEARCH BADGES

This article has earned an Open Data Badge for making publicly available the digitally‐shareable data necessary to reproduce the reported results. The data is available at: https://doi.org/10.5061/dryad.5hqbzkh3m.

## Supporting information

Supplementary Material

## Data Availability

Data are archived in the repository *Dryad* at: https://doi.org/10.5061/dryad.5hqbzkh3m.
