# Supplementary material for: Fine‐scale habitat selection by sympatric Canada lynx and bobcat
Source: Ecol Evol. 2020 Aug 17;10(17):9396–409. doi: 10.1002/ece3.6626 (PMC7487242; doi:10.1002/ece3.6626)
Supplement: Supplementary file 1 — Supplementary Material [file ECE3-10-9396-s001.pdf]

Appendix

Table A1. AICc for the full models of Canada lynx (*Lynx canadensis*) and bobcat (*Lynx rufus*) habitat selection on the north shore of Lake Huron, Ontario, analyzed using conditional logistic regression. 10, 20, 30, 40, and 50 m represent the five different buffer scales used to measure coniferous forest and immature forest. Predictors in the full models included snow depth, snow hardness, snowshoe hare, deer, squirrel, grouse, coniferous forest, and immature forest.

Species	10 m	20 m	30 m	40 m	50 m
Canada lynx	40.23	39.10	40.69	41.01	43.32
Bobcat	39.98	39.86	40.13	42.57	44.64

Table A2. Matrix of the Pearson correlations of the amounts of coniferous forest associated with used and unused Canada lynx (*Lynx canadensis*, top triangle) and bobcat (*Lynx rufus*, bottom triangle) paths on the north shore of Lake Huron, Ontario, measured with five different buffer scales. For all correlations, $p<0.05$. For all buffer scales, $n=60$.

	10 m	20 m	30 m	40 m	50 m
10 m		0.946	0.961	0.948	0.947
20 m	0.978		0.977	0.989	0.970
30 m	0.984	0.987		0.988	0.982
40 m	0.978	0.988	0.995		0.987
50 m	0.963	0.976	0.990	0.995	

Table A3. Matrix of the Pearson correlations of the amounts of immature forest associated with used and unused Canada lynx (*Lynx canadensis*, top triangle) and bobcat (*Lynx rufus*, bottom triangle) on paths on the north shore of Lake Huron, Ontario, measured with five different buffer sizes. For all correlations, $p<0.05$. For all buffer scales, $n=60$.

	10 m	20 m	30 m	40 m	50 m
10 m		0.981	0.988	0.984	0.956
20 m	0.981		0.988	0.990	0.954
30 m	0.985	0.985		0.997	0.971
40 m	0.952	0.974	0.981		0.973
50 m	0.970	0.974	0.992	0.992	

Table A4. Matrix of the Pearson correlations between predictors (*n*=60) used to model Canada lynx (*Lynx canadensis*) (upper triangle) and bobcat (*Lynx rufus*) (lower triangle) habitat selection on the north shore of Lake Huron, Ontario using conditional logistic regression. Coniferous forest and immature forest were measured with a 20 m buffer size.

	Snow depth	Snow hardness	Snowshoe hare	Deer	Squirrel	Grouse	Coniferous	Immature forest
Snow depth		0.189	-0.045	0.067	0.030	-0.142	0.173	0.001
Snow hardness	0.374		-0.114	0.250	-0.040	-0.201	0.170	0.059
Snowshoe hare	0.275	0.015		-0.071	-0.022	0.174	0.108	-0.175
Deer	-0.055	0.178	0.119		0.048	-0.088	0.189	0.169
Squirrel	-0.309	-0.459	0.168	-0.127		0.075	-0.245	0.012
Grouse	-0.145	-0.087	0.126	-0.086	0.083		-0.070	-0.173
Coniferous forest	-0.093	0.124	-0.285	0.153	-0.173	-0.111		0.379
Immature forest	0.213	0.098	0.396	0.093	-0.132	-0.057	-0.036	
